# Ultra-Performance Liquid Chromatography-Ion Mobility Separation-Quadruple Time-of-Flight MS (UHPLC-IMS-QTOF MS) Metabolomics for Short-Term Biomarker Discovery of Orange Intake: A Randomized, Controlled Crossover Study

**DOI:** 10.3390/nu12071916

**Published:** 2020-06-29

**Authors:** Leticia Lacalle-Bergeron, Tania Portolés, Francisco J. López, Juan Vicente Sancho, Carolina Ortega-Azorín, Eva M. Asensio, Oscar Coltell, Dolores Corella

**Affiliations:** 1 Research Institute for Pesticides and Water (IUPA), Universitat Jaume I, 12071 Castellón, Spain; mlacalle@uji.es (L.L.-B.); tania.portoles@qfa.uji.es (T.P.); lopezf@uji.es (F.J.L.); sanchoj@uji.es (J.V.S.); 2 Department of Preventive Medicine and Public Health, School of Medicine, University of Valencia, 46010 Valencia, Spain; Carolina.Ortega@uv.es (C.O.-A.); eva.m.asensio@uv.es (E.M.A.); 3 CIBER Fisiopatología de la Obesidad y Nutrición (CIBEROBN), Instituto de Salud Carlos III, 28029 Madrid, Spain; oscar.coltell@uji.es; 4 Department of Computer Languages and Systems, Universitat Jaume I, 12071 Castellón, Spain

**Keywords:** orange intake, metabolomics, ion mobility, biomarkers

## Abstract

A major problem with dietary assessments is their subjective nature. Untargeted metabolomics and new technologies can shed light on this issue and provide a more complete picture of dietary intake by measuring the profile of metabolites in biological samples. Oranges are one of the most consumed fruits in the world, and therefore one of the most studied for their properties. The aim of this work was the application of untargeted metabolomics approach with the novel combination of ion mobility separation coupled to high resolution mass spectrometry (IMS-HRMS) and study the advantages that this technique can bring to the area of dietary biomarker discovery, with the specific case of biomarkers associated with orange consumption (*Citrus reticulata*) in plasma samples taken during an acute intervention study (consisting of a randomized, controlled crossover trial in healthy individuals). A total of six markers of acute orange consumption, including betonicines and conjugated flavonoids, were identified with the experimental data and previous literature, demonstrating the advantages of ion mobility in the identification of dietary biomarkers and the benefits that an additional structural descriptor, as the collision cross section value (CCS), can provide in this area.

## 1. Introduction

Currently, one of the main limitations of nutritional epidemiology is the difficulty involved in measuring dietary intake [1]. In observational studies carried out on a large number of participants, the most commonly applied tools for estimating dietary intake are mainly based on self-reporting, including food frequency questionnaires (FFQs) for the assessment of regular consumption (usually one-year), or 24-h recalls for one-day assessment. However, such methodologies for data collection may contain substantial recall bias and other systematic or random errors that may have a large effect on the estimated food intake and, furthermore, on subsequent associations between food intake and the diseases studied [2,3]. Although in recent years, there has been an improvement in increasing the validity and precision of food questionnaires thanks to the use of new information technologies [4,5], these instruments are still biased and additional information based on objective intake biomarkers is needed [6,7]. Biomarkers of food intake are promising tools to provide more objective food consumption measurements [6,7,8,9]. Therefore, a major challenge nowadays for nutritional epidemiology and precision nutrition is to identify novel and valid biomarkers [10]. Metabolomics has opened up new opportunities for food intake biomarker discovery through metabolic profiling of biological samples (plasma, urine, etc.), following the intake of specific foods, meals, or diets [10,11]. Nevertheless, to date, there are very few validated biomarkers of food intake, and more research is urgently needed. In a consensus paper, Dragsted et al. [10] outlined an optimal and reproducible validation process to systematically and critically assess the validity of candidate food intake biomarkers. A consensus-based procedure was used to provide and evaluate a set of the most important criteria for systematic validation of these biomarkers [10,12]. Similar conclusions were reached in a National Institute of Health (NIH) organized workshop on “Omics Approaches to Nutritional Biomarkers” in Bethesda, attended by researchers from the United States, Canada, and several European countries [13]. According to these recommendations, acute interventions trials where participants consume specific amounts of a test food in a single meal are the best approach as the first step in dietary biomarker discovery [10,11,12,13]. In this approach, baseline and postprandial biological samples are collected for analysis and for identifying potential biomarkers. For these dietary interventions, the use of crossover studies is an efficient design having less bias and requiring less sample size than randomized parallel group intervention trials [14]. In crossover studies, participants receive all interventions in consecutive periods, which means that the influence of individual variation is minimized. The metabolites are measured after every period. In these studies, the use of a single food, facilitates a more specific investigation of biomarkers, since the number of potential metabolites is simplified in comparison with a full dietary pattern. Despite this simplification, identifying metabolites for dietary intake is not always easy, even if only one food is administered [15]. Small, short-term feeding studies that yield candidate biomarkers may be followed by validation and testing of biomarker performance in large cohorts, repeating the process with required corrections, until an ideal biomarker performance is achieved. Currently, oranges are one of the foods for which it has been shown that there are good metabolomic biomarkers of intake [16,17,18]. Specifically, an excellent example of such a biomarker for citrus fruit intake is proline betaine (*N*,*N*-dimethyl-L-proline), also known as stachydrine [16,17,18,19,20]. This biomarker was first discovered in a small crossover trial [19], and later validated in several intervention and cohort studies [16,18,20,21,22,23,24]. Despite the high statistically significant associations found between citrus intake and proline betaine levels (both in plasma and/or in urine), correlation coefficients ranged between 0.4 and 0.8 depending on the study, suggesting that other additional biomarkers for citrus intake can add more information in precision nutrition. Some studies reported additional biomarkers for citrus fruit intake including trans-4-hydroxy-L-proline betaine (betonicine), N-methyl-L-proline and synephrine, among others [17,25,26,27,28,29,30]. However, with improvement in techniques and instruments, more biomarkers can be discovered. Moreover, it has been suggested that, whereas proline betaine is a good biomarker for the intake of any citrus product, synephrine can be used as a reliable additional biomarker with high specificity for orange intake [30].

Aside from specific compounds, in metabolomics the analytical platform used as well as the targeted or untargeted approaches, may have a role to play in identifying metabolites [31], each of these having a number of advantages and disadvantages [31]. Food is usually a complex and diverse mixture of components that could present a metabolic potential or not. To analyze these compounds, there is a need for robust, efficient and sensitive methodologies along with powerful analytical technologies [32,33]. Metabolomic fingerprinting is the global screening approach (untargeted method) used for low molecular mass compounds (<1500 Da), for classifying the samples based on the metabolic profile or “fingerprints” that change in response to external (environmental, diet…) or internal (genetic, disease…) perturbations with the final goal of identifying or discriminating between metabolites [15,33,34].

Due to the large amount of compounds that are joined in the metabolome, with a large variety of physicochemical properties, in recent years the combination of separation techniques such as gas or liquid chromatography (GC and LC respectively) with high resolution accurate mass spectrometry (HRMS) are appearing to be less expensive and more sensitive alternative approaches than nuclear magnetic resonance (NMR) [35], which has been the previous technique of choice [36]. Because of the physical properties of most biofluids, GC is not usually chosen in this field because the analytes (usually polar and not very volatile) often requires derivatization to improve thermal stability for GC, requiring more laborious sample treatment [37,38]. Due to the aqueous composition of biofluids (such as blood, urine, sweat), LC has been widely used for metabolomics in bioanalytical studies. Furthermore, a wider range of separation mechanisms due to the variety of stationary phases is available, so that complex sample preparation is not usually required, and shorter separation run times are involved [39].

Therefore, hyphenation LC-HRMS is widely employed in untargeted metabolomic approaches. Moreover, the recent introduction of ion mobility separation (IMS) coupled to HRMS Q-TOF provides additional structural information, so achieving a better characterization of biomakers [40,41,42]. IMS is a gas-phase separation technique based on the time that an ion takes to cross a drift tube filled with an inert gas under low-field conditions. The drift time (DT) of each ion is dependent on its individual size, shape and charge [43,44]. DT can be directly converted into a collision cross section value (CCS, Å^2^), which represents an additional separation dimension based on the compound chemical structure and 3-dimensional conformation [44,45]. The LC-IMS-HRMS combination provides an extra separation dimension through CCS to the retention time (RT), accurate mass (*m*/*z*) and intensity (I), thus obtaining four-dimensional matrix data.

Therefore, the main aim of this study was to perform an exploratory untargeted metabolomic study using UHPLC-IMS-HRMS to find out short-term plasma biomarkers of orange consumption, as well as to explore the promising improvements that IMS in combination with LC-HRMS could achieve in the untargeted metabolomic field in an acute crossover intervention trial using multivariate analysis (PCA, PLS-DA and OPLS-DA) to highlight the most relevant short-term markers of orange intake.

## 2. Materials and Methods

### 2.1. Subjects and Study Design

We carried out a randomized, controlled crossover study on 30 healthy subjects (aged 25.0 ± 2.8 years, 8 females and 22 males), recruited in Valencia, Spain. After a minimum of 8 h fasting, participants were randomly allocated to eat 500 g of peeled oranges (*Citrus reticulata, Clementine variety)*) or an isocaloric (same energy as the oranges) solution of sucrose in water. No other food was allowed for 4 h (a standard time-point for acute human studies using plasma samples). At the start and after 4 h, blood samples were taken. Subjects were randomized to start either in the intervention or the control groups in order to prevent the influence of period-effects. In addition, a wash-out period was considered. After a one-week break the two groups swapped over and the corresponding intervention was undertaken. The crossover study was registered as ISRCTN17330010. Anthropometric, clinical, lifestyle and biochemical data were obtained by standardized techniques and questionnaires as previously described [46]. Plasma was obtained and stored at −80 °C for subsequent metabolomics determinations. Subjects provided written informed consent and the study protocol and procedures were approved according to the ethical standards of the Helsinki Declaration and by the Human Research Ethics Committee of the University of Valencia, Valencia (reference number: H1425917369903).

### 2.2. Chemicals and Reagents

LC-MS grade methanol (MeOH) and LC-MS grade acetonitrile (ACN) were purchased from Sharlab (Barcelona, Spain), as well as Formic acid (HCOOH) eluent additive for LC-MS and reagent grade ammonium acetate (NH_4_Ac). Milli-Q water purification system (Millipore Ltd., Bedford, MA, USA) was used to obtain HPLC-grade water. Leucine-enkephalin (mass-axis calibration) was purchased from Sigma-Aldrich (Saint Louis, MO, USA).

### 2.3. Sample Treatment

Prior to analysis, the blood plasma samples were thawed at room temperature and vortexed. A total of 400 µL of ACN was added to 100 µL of sample. The supernatant was collected after centrifuging 12,000× *rpm* and a radius of 5.5 cm, 8855 *g* (RCF), for 10 min at 4 °C, and divided into 3 aliquots: two vials of 100 µL were stored at −30 °C, one vial of 200 µL was stored at −80 °C. Moreover, 20 µL of each sample were pooled and mixed to generate the Quality Control sample (QC). This is assumed to provide a representative average sample formed by a pool equivalent aliquot of all final sample extracts.

In order to compensate/reduce instrumental drift, samples were randomly injected into the UHPLC-IMS- QTOF MS system. QC was used for both column stabilization purposes (by 10 QC injections at the beginning of each sample batch) and, following the injection of every 10 samples, to control possible instrumental drift throughout the sequence.

### 2.4. Instrumentation

Ultra-high performance liquid chromatography (UHPLC) with a ACQUITY UHPLC I-Class system (Waters, Milford, MA, USA) was coupled to a VION^®^ IMS QTof (Waters, Manchester, UK), ion mobility hybrid Quadrupole Time-of-Flight (IMS-QTOF) High Resolution Mass Spectrometer (UHPLC-IMS-HRMS) using a electrospray ionization interface operating in both positive (ESI+) and negative (ESI−) mode. Equipment control and data acquisition and processing were performed using UNIFI software (V.1.8.2, Waters, Manchester, UK).

#### 2.4.1. UHPLC Conditions

Two LC separation procedures were employed to cover a wider range of compound polarities. To separate the medium to nonpolar molecules, Reversed Phase Liquid chromatography (RP) was used with a CORTECS^®^ C18 fused-core 2.7 μm particle size analytical column 100 × 2.1 mm (Waters), whereas a CORTECS^®^ HILIC fused-core 2.7 μm particle size analytical column 100 × 2.1 mm (Waters) was employed for Hydrophilic Interaction Liquid Chromatography (HILIC) to separate the polar molecules. 0.3 mL/min flow rate, 40 °C column oven temperature and 1 μL sample injection volume were selected for both types of liquid chromatography and both ionization modes.

The RP-LC gradient elution was performed using mobile phases A = H_2_O and B = MeOH, both with 0.01% of HCOOH. The gradient changed from 10% B at 0 min to 90% B at 14 min, 90% B at 16 min, and 10% B at 16.01 min, with a total run time of 18 min. The same gradient was employed for both ESI+ and ESI− ionization modes.

For HILIC separation, mobile phases A = ACN:H_2_O (95:5, *v*/*v*) and B = H_2_O, both with 0.01% HCOOH and 10 mM NH_4_Ac, were employed. The gradient started with 2% B until 1.50 min, 15% B at 2 min, 50% B at 6 min, 60% B at 7.50 min and finally 2% B at 7.51 min, with a total run time of 10 min. This gradient was the same for both ESI+ and ESI− ionization modes.

#### 2.4.2. IMS-QTOF Set Up

The capillary voltage was set at 0.7 kV and 2.00 kV for positive (ESI+) and negative (ESI−) electrospray ionization mode, respectively. Nitrogen was used as the desolvation gas, nebulizing gas and collision gas. The source temperature was set to 120 °C and desolvation gas to 550 °C with a flow rate of 1000 L/h. The mass spectrometer was operated in ion mobility (HDMS^E^) mode for acquisition in both polarities. In HDMS^E^ experiments, two acquisition functions were acquired simultaneously over an *m*/*z* range of 50–1000 Da and a scan time of 0.3 s. Low Energy function (LE), with a fixed collision energy of 6 eV, and High Energy function (HE) with a collision energy ramp from 28 to 56 eV was set.

Calibrations of mass axis and DT were performed monthly with the “Major Mix IMS/T of calibration kit” supplied by the vendor (Waters), infused at a flow rate of 20 µL/min for both positive and negative mass calibrations as well as CCS calibration.

For automated mass measurement, a Leucine-Enkephalin solution (100 ppb) in ACN:H_2_O (50:50, *v*/*v*) at 0.01% HCOOH was pumped at 20 µL/min through the lock-spray needle and measured every 5 min (ensuring a measurement at the beginning, in the middle and at the end of the chromatogram), with a scan time of 0.3 s. The (de)protonated molecule of leucine-enkephalin, at *m*/*z* 556.27658 in ESI+ and *m*/*z* 554.26202 in ESI− was employed to recalibrate the mass axis and ensure the robust accurate mass measurement along runs.

### 2.5. Data Processing and Statistical Analysis

The VION instrument data (.uep, UNIFI, Waters) were imported to Progenesis QI (V.2.3, NonLinear Dynamics, Waters, Newcastle, UK). Then, the software automatically performs the retention time alignment, with the QC samples as reference (except for the first 9 QC injections used for column stabilization); the 4D peak picking (based on the intensity, *m*/*z*, retention time and DT) and response normalization. The peak picking conditions were set as follows: all runs, limits (automatic), sensitivity (automatic, level 2), chromatographic peak width (minimum peak width of 0.1 min), and retention time limits (0.3 to 17 min and 0.3 to 9 min, for RP and HILIC respectively). To apply the deconvolution tool, the selected adducts ions forms [M+H]^+^, [M-H_2_O+H]^+^, [M+Na]^+^ and [M+K]^+^ were selected for positive ionization analysis and [M-H]^−^, [M-H_2_O-H]^−^, [M+Cl]^−^, [M+FA-H]^−^ for negative ionization analysis. Samples were divided into 4 groups (Orange t = 0 h, Orange t = 4 h, Isocaloric Beverage (IB) t = 0 h, IB t = 4 h) in the “Experimental Design Setup”, following the “Within-subject Design” comparison, where not only are the groups each sample belongs to specified to the software, but also the subject from which it comes. The software will then perform a repeated measures ANOVA, to reduce the individual differences.

The processed data were then directly exported to EZinfo (V.3.0, Umetrics, Sweden) for multivariate statistical analysis. First, Principal Component Analysis (PCA), an unsupervised analysis, was applied to ensure the correct grouping of the QC samples in the center of the plot after normalization and the absence of outliers. Then, Partial Least Square–Discriminant Analysis (PLS-DA) was conducted to maximize the separation between the groups. Ultimately, an Orthogonal PLS-DA (OPLS-DA) was carried out to highlight the most robust markers (threshold P[corr] ≥ |0.6|).

### 2.6. Elucidation Workflow

The accurate mass and retention time of the most significant markers from the OPLS-DA were obtained from the feature table and they were checked and sought in the raw data (by UNIFI Platform). The compounds were tentatively elucidated with the aid of an elemental composition calculator (UNIFI, V1.8.2, Waters). The DT filtered HE spectra of the target feature was searched in reliable mass spectra data bases (HMDB, MetLin, MassBan) or were compared to in-silico fragmentation spectra (MetFrag) with subsequent searches through general chemical data bases such as Chemspider and PubChem.

The final identity could only be confirmed by comparing the retention time, fragmentation and CCS with a commercially available standard. When not available, CCS values were predicted by means of our CCS prediction model [45] aimed at providing additional identification confidence.

## 3. Results

### 3.1. Participants and Experimental Setup

Table 1 shows the general characteristics of the study participants. A total of 30 healthy subjects (97% nonsmokers) were analyzed in the intervention study and all of them completed both the dietary intervention with oranges and the control group (isocaloric intervention with sucrose) at baseline and after 4 h. Plasma samples were obtained twice at baseline and after 4 h and analyzed for metabolomics biomarkers of orange intake.

Regarding sample treatment, the only step taken was deproteinization with ACN to eliminate the macromolecules (nucleic acids and proteins) present in the plasma and, thereby, avoid possible interferences in the metabolomic analysis and so focus on the low-weight molecules (metabolites). The sample treatment must be as low selective as possible to cover a larger range of compounds. The supernatant was directly injected in the UHPLC-IMS-QTOF MS system. The samples extracts were injected in randomized order to avoid bias in the methodology and QC was injected every 10 samples for normalization and instrumental drift control.

Owing to the aqueous nature of the plasma samples, LC was the most convenient chromatographic separation technique. Furthermore, due to the high sensitivity of the instrument employed, only a low volume injection was possible (1 µL). This allowed the samples extracts (ACN:H_2_O, 80:20 *v*/*v*) to be directly injected not only in HILIC, where the initial chromatographic conditions were predominantly in the organic phase; but also in RP, without the need to evaporate and redissolve in a more suitable solvent mixture (predominantly aqueous medium). RP and HILIC were employed to detect as many compounds as possible, in positive (pos) and negative (neg) ionization modes. RP (C18 column) is more suited to nonpolar hydrophobic molecules and HILIC (silica-based column) for polar hydrophilic compounds. Finally, we obtained four data sets for the samples under study (RP pos, RP neg, HILIC pos and HILIC neg).

All data were acquired in HDMS^E^, where, apart from the DT (ms) measurement by ion mobility, LE and HE spectra were also acquired simultaneously, obtaining precursor ion information and full-scan accurate mass fragmentation information, respectively. Therefore, at the end of the analysis, a four-dimensional data matrix was obtained, which allowed each feature to be characterized by means of the retention time from chromatographic separation, the intensity, the CCS calculated from the DT together with the accurate mass and fragmentation spectra. Moreover, the cleaned HE MS spectra afforded by the DT separation enhanced the structural elucidation since it only shows the fragments that have been generated from a “precursor” ion with a given DT. This is because the ion mobility separation prior to the fragmentation ensures that the precursor ion shares the same DT as its fragments on the three-dimensional plots, allowing one to align the feature and its fragments and reduce the interference of co-eluting components, thus rendering a HE spectra of MS/MS quality.

### 3.2. Data Processing and Analysis

The four data sets (RP pos, RP neg, HILIC pos and HILIC neg) were acquired with UNIFI software (Waters, UK) and the raw data were exported to *.uep format (unifi export package). To the best of our knowledge, there is no program but Progenesis QI (NonLlinear Dynamics, Waters, Uk) able to interpret this data format and process four-dimensional data (RT, *m*/*z*, area and CCS) for -omics purposes. The processing workflow in Progenesis QI starts with data import, followed by retention time alignment, peak picking and normalization. The alignment score values for all runs were higher than 85% and normalization was performed using all compounds. The peak picking resulted in the detection of 6951, 6238, 5283 and 4479 ions in RP pos, RP neg, HILIC pos and HILIC neg, respectively. According to the adduct ions specified, the deconvolution tool allowed us to group the features coming from the same compound and annotate them under a single label (*xx.xx_yyy.yyyyn, xx.xx* being the retention time in minutes and *yyy.yyyy* the exact neutral mass when more than one adduct ion is found for the same compound; or *xx.xx_zzz.zzzzzm/z, zzz.zzzz* being the exact mass of the single ion found).

**Figure 1:**
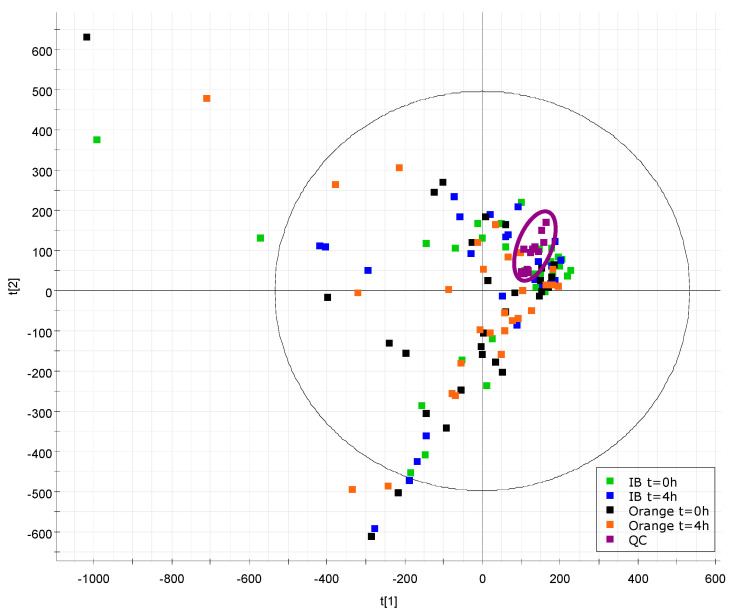
PCA score plot component 1 vs. component 2 of Hydrophilic Interaction Liquid Chromatography (HILIC) in positive ionization mode, explaining the 20.6% and 17.7% of the variance, respectively. The purple points (14 QC samples) are grouped and centered in the plot. Four plasma samples were obtained from each of the 30 participants obtaining 30 samples per group. A total of 120 plasma samples were analyzed. The samples obtained at t = 0 h and t = 4 h after the intake of an isocaloric beverage (IB) and the samples obtained at t = 0 h and t = 4 h after orange intake are colored in green, blue, black and orange, respectively.

PCA was applied to each data set, so providing a nonsupervised exploratory visualization of the results to detect possible outliers and to ensure that the differences between the groups were not coming from the instrumental drift over time or error in the data treatment. For this purpose, QC injections were injected at the beginning of the batch for column conditioning and after every 10 samples. The QCs (*n* = 14 per data set) should be grouped in the center of the PCA plots, as they behave as an “average sample” (pool of all the samples analyzed) and thus demonstrate the correct acquisition of the data, as well as that the differences between the groups are not caused by instrumental processing. Figure 1 shows the PCA of the HILIC pos data set, where noninherent differentiation between the group of samples can be observed. Although, the grouping of the QC samples near to the center of the plot proves the proper performance of the analytical system along the run. PCA plots of the data sets HILIC neg, RP pos and RP neg are shown in Figures S1–S3 respectively.

As this was a crossover trial, control samples of each participant were collected and a “Within-subject Design” comparison performed. In this type of experimental design, where samples of each participant are taken in different conditions, not only must the time the sample was taken be specified, but also which subject it came from. In these cases, standard ANOVA is not appropriate because data violates the independence ANOVA assumption and a repeated measures ANOVA should be applied. The data were filtered by means of repeated measures ANOVA *p*-value ≤ 0.05 to reduce the individual differences and focus on the potential markers. Thus, the data matrices were reduced to 262, 302, 298 and 149 relevant features in RP pos, RP neg, HILIC pos and HILIC neg, respectively. These reduced data sets were joined into a single file (1011 features data matrix).

**Figure 2:**
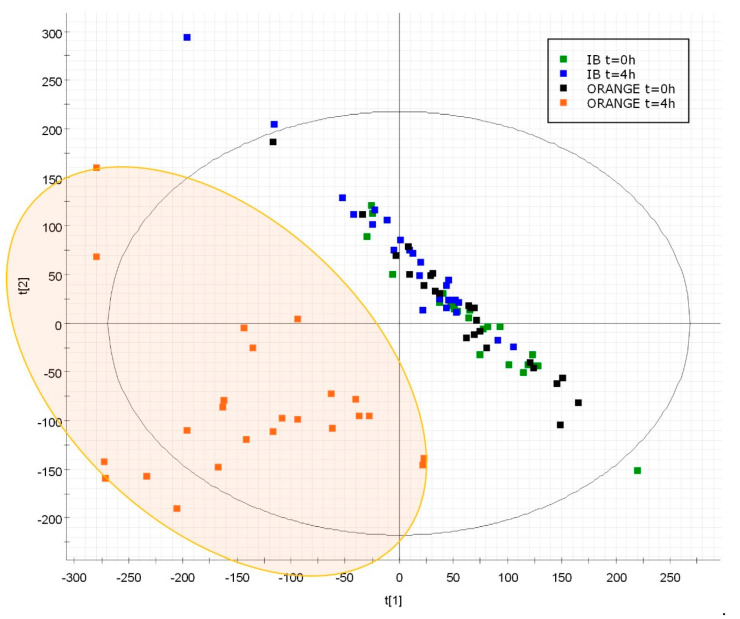
PLS-DA score plot based on the features with repeated measures ANOVA *p*-value ≤ 0.05. A 2D vision component 1 vs. component 2 with 24.5% and 13.8% of the variance explained, respectively. The samples obtained at t = 0 h and t = 4 h after the intake of an isocaloric beverage (IB) and the samples obtained at t = 0 h and t = 4 h after orange intake are colored in green, blue, black and orange, respectively.

The next step was to apply a supervised multivariate statistical method by means of PLS-DA modeling to highlight the differences between the preselected groups. This analysis was applied to the compendium of the reduced data sets per ANOVA. Figure 2 shows a remarkable discrimination between samples taken after orange consumption and those obtained in fasting or after isocaloric beverage intake (with 38.3% of the variance explained with two components).

**Figure 3:**
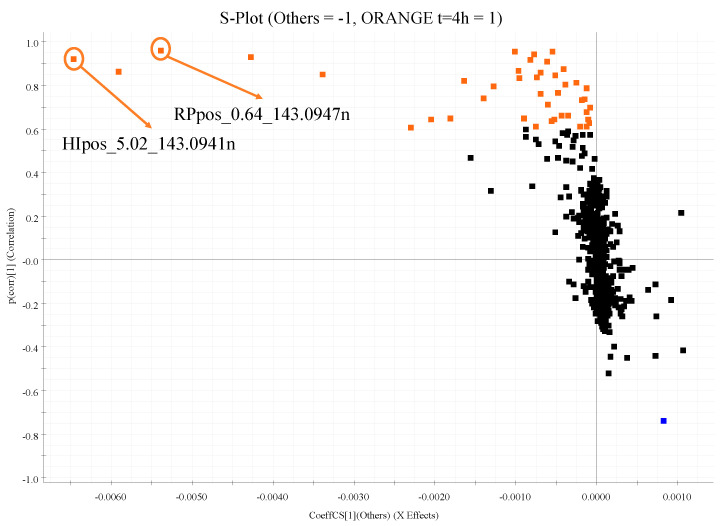
S-Plot from the OPLS-DA where features with repeated measures ANOVA *p*-value ≤ 0.05 are represented. Tentative markers with a P[corr] higher than 0.6 are highlighted in orange and lower than −0.6 in blue.

Moreover, these last three groups are mixed and behave in a similar way. Thus, an OPLS-DA was applied contrasting the samples collected 4 h after orange intake vs. the other samples (Figure S4 shows the scatter plot of the OPLS-DA). From OPLS-DA an S-Plot was generated (Figure 3), each feature being assigned a number between −1 and 1 according to their discrimination power between the two groups called P[corr]. Consequently, the most relevant features in the discrimination are in the extreme parts of the plots: those with a higher presence in the orange intake sample have a P[corr] near to 1 and those significant for the other group have a P[corr] near −1. To ensure the validity of these features as possible markers, a PCA analysis was conducted with only the 43 highlighted features in the S-Plot as variables. As can be noticed in Figure S5, the samples obtained before and after the isocaloric beverage and the samples before the orange consumption are clustered and completely separated from the samples obtained 4 h after the orange intake. Figure 3 shows that, from the total of 1011 features, only 43 from all data sets with a repeated measures ANOVA *p*-value ≤ 0.05 were highlighted as discriminatory between the samples after orange consumption and the group of composite samples without orange intake with a P[corr] ≥ |0.6|. Nevertheless, the list of possible markers could be reduced still further to seven features for various reasons. For example, two features higher in the orange sample with the same exact mass and the same CCS (127.18 Å^2^) but different retention time (HIpos_5.02_143.0941n and RPpos_0.64_143.0947n, with P[corr] = 0.96 and 0.92, respectively) corresponded to the same compound, but detected under HILIC and RP separation. In other cases, some types of adducts (such as dimers) had not been specified for the Progenesis QI deconvolution step, and therefore these adducts could appear as independent features in the statistics.

### 3.3. Elucidation Process

**Figure 4:**
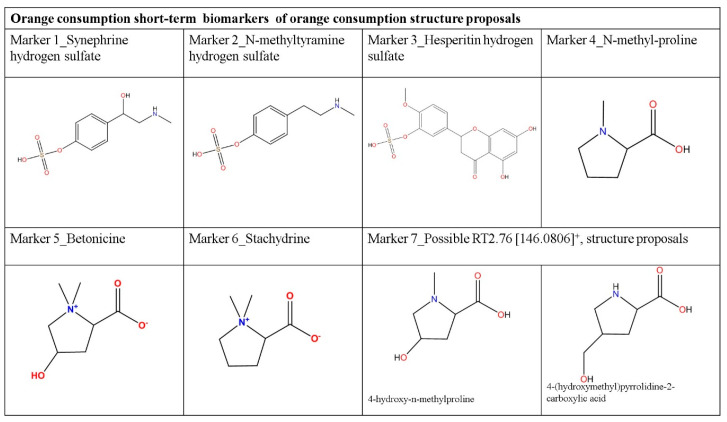
Tentative structures and identity of the makers elucidated.

A total of 7 statistically representative compounds were tentatively identified (Figure 4, Table 2) as early markers of orange consumption in plasma. In order to accomplish the elucidation, the HDMS^E^ spectra were studied to determine the candidate identity based on mass accuracy and both parents and fragment ions.

**Figure 5:**
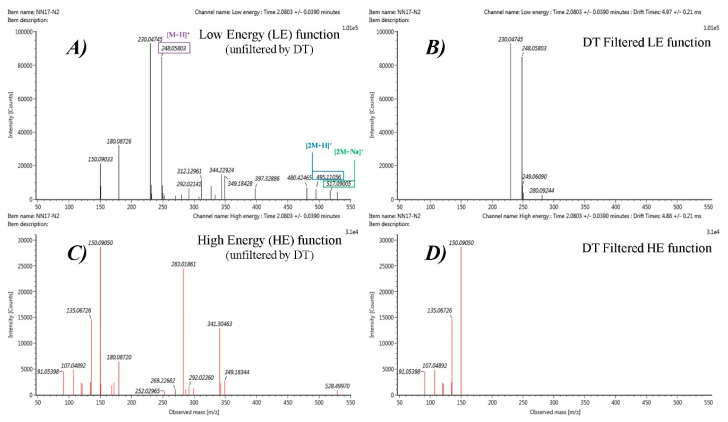
HDMS^E^ spectra from HILIC pos analysis for Marker 1 with and without DT filtering: (**A**) LE function (without DT filtering), (**B**) DT filtered LE function, (**C**) HE function (without DT filtering), and (**D**) DT filtered HE function.

Marker 1 elucidation workflow was selected as an illustrative example of the elucidation process. Figure 5 shows the differences obtained in the mass spectra when it is filtered or not by the DT of the parent ion (4.97 ± 0.21 ms). The ion mobility separation prior to the fragmentation implies that both parent and fragment ions will have the same recorded DT. Hence, it is possible to filter the target ion by DT and obtain cleaner spectra without interfering ions appearing at the same retention time for the LE spectrum and without co-elutant fragments in the HE spectrum. The possibility of visualizing only “product” ions derived from a specific “precursor” ion, allows us to obtain a quasi-MS^2^ spectrum, without the need for reinjecting the samples.

However, information was also obtained from the unfiltered spectra. Since the adduct ions are formed before ion mobility separation, their DT could be different, and therefore they would not be shown in the DT filtered spectrum. In the case of Marker 1 (HIPOS_2.17_247.0510n, P[corr] = 0.64), as can be seen in Figure 5A, the dimers [2M+H]^+^ and [2M+Na]^+^ are also present, as dimers were not selected as possible “adduct” ions in the Progenesis QI deconvolution, but were assigned as independent features. These features were also highlighted as discriminant in the OPLS-DA with a P[corr]>0.6.

**Figure 6:**
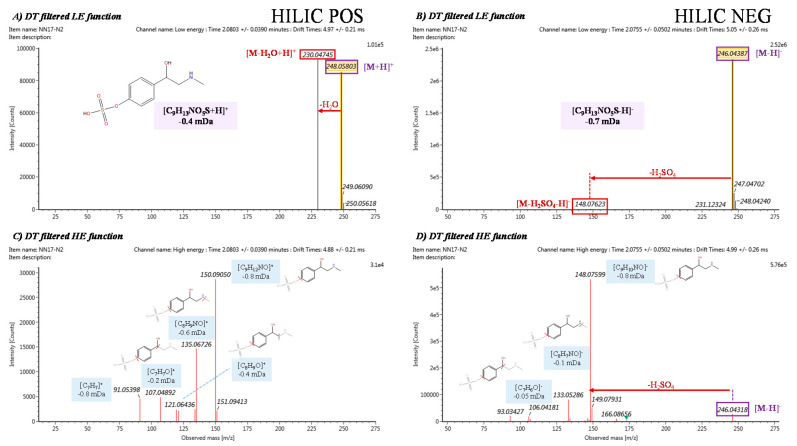
DT filtered HDMS^E^ obtained for Marker 1: (**A**) LE function in HILIC pos analysis, (**B**) LE function in HILIC neg analysis, (**C**) HE function in HILIC pos analysis and (**D**) HE function in HILIC neg analysis. A proposed structure and fragmentation are shown.

Marker 1 was also found in HILIC neg analysis (HINEG_2.22_246.0434m/z) with different ion forms. The extracted ion chromatograms for the ions and dimers founded corresponding to this marker are shown in Figure S6, and all of them are highlighted as markers. The most likely elemental composition for this marker was found to be C_9_H_17_NO_5_S (error: −0.4 and −0.07 mDa in positive and negative HILIC analysis, respectively). The mean retention time across the samples was 2.2 min and CCS value 158.27 Å^2^ and 155.40 Å2 for protonated and deprotonated ion, respectively. Figure 6 shows the LE and HE spectra obtained in HILIC pos and HILIC neg analysis, filtered by the DT of the protonated and deprotonated ion, respectively.

In the HILIC pos LE function, an in-source fragment *m*/*z* 230.0476 ([C_9_H_12_NO_4_S]^+^, error: −0.7 mDa) corresponding to a neutral loss of H_2_O (not very specific fragment) can be seen. From the HILIC neg LE function is obtained a more specific in-source fragment *m*/*z* 148.0762 ([C_9_H_10_NO]^−^, error: −0.7 mDa) corresponding to the neutral loss of H_2_SO_4_, a typical loss of phase II sulfate conjugated metabolites. This ion is also present in the HILIC neg HE spectrum as a product ion, with *m*/*z* 133.0529 ([C_8_H_7_NO]^−^, error: −0.1 mDa) assigned as consecutive neutral loss of CH_3_, followed by neutral loss of HCN with *m*/*z* 106.0418 ([C_7_H_6_O]^−^, error: −0.05 mDa). These ions were also present in HILIC pos HE spectra in their corresponding positive adducts *m*/*z* 150.0905 ([C_9_H_12_NO]^+^, error: −0.8 mDa), *m*/*z* 135.0673 ([C_8_H_9_NO]^+^, error: −0.6 mDa) and *m*/*z* 107.0418 ([C_7_H_7_O]^+^, error: −0.2 mDa), among other product ions. After the automated searching in databases with the UNIFI (Waters) tool, in-silico fragmentation tools and spectra comparison with data bases as HMDB if it is available, Synephrine hydrogen sulfate, phase II sulfate conjugated metabolite of synephrine, was the most likely tentative identification, and this match was supported by the fragmentation spectra.

Synephrine is a citrus compound and commonly present in dietary supplements (coming from citrus extracts) used in weight loss, and therefore its metabolism and toxicity has been extensively studied [47]. Moreover, it has previously been reported as a biomarker of citrus intake in urine, along with other metabolites of other flavonoids citrus compounds as Marker 2 and 3, tentatively identified as phase II sulfate conjugated metabolites of N-Methyltyramine and Hesperitin, respectively (Table 2 and Table 3). These compounds have been previously studied due to their antioxidants and anti-inflammatory properties [48,49]. Moreover, hesperitin phase I/II conjugates have already been described as major metabolites of other citrus containing the flavonoids hesperidin and naringenin [50,51].

.

**Figure 7:**
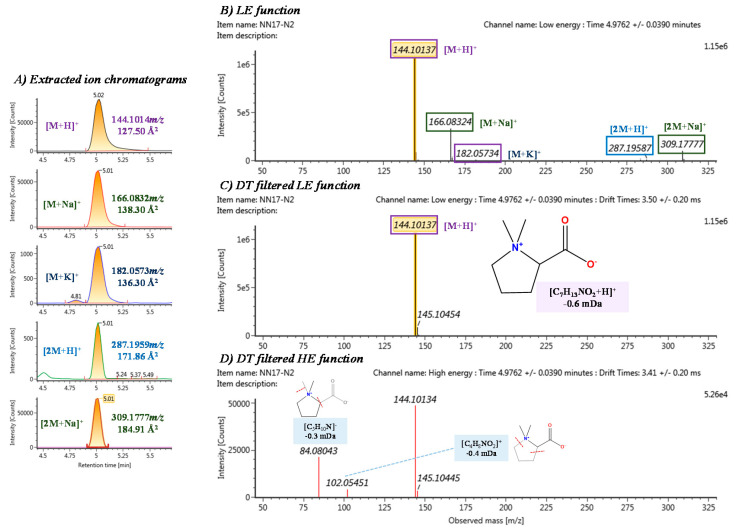
Elucidation of marker 6 based on chromatograms and HDMS^E^ data obtained from HILIC pos analysis: (**A**) extracted ion chromatograms of all adduct and dimers found, along with the experimental *m*/*z* and CCS, (**B**) LE function, (**C**) DT filtered LE function and (**D**) DT filtered HE function. A proposed structure and fragmentation are shown.

In the case of Marker 6, Figure 7 shows the extracted ion chromatograms and HDMS^E^ spectra in HILIC pos analysis. This marker was also found in the dead volume in RP pos due to its high polarity, corresponding to the features HIpos_5.02_143.0941n and RPpos_0.64_143.0947n highlighted in Figure 3, among other features corresponding to dimers. Similar behavior occurred in Marker 5. These two markers were the ones with higher P[corr] and abundancy. Therefore, they are the most differentiating markers present in the plasma samples 4 h after orange consumption (Table 2). These markers were tentatively elucidated as Betonicine and Stachydrine (proline betaine). These betaines have been extensively reported as a citrus intake biomarker in blood and urine (Table 3).

Indeed, stachydrine is one of the most commonly used dietary biomarkers for dietary assessment of citrus intake and it has even been related to the amount consumed [18,20]. Betonicine has also been detected in several studies, as well as other betonicines as Marker 4, tentatively elucidated as N-methyl-proline, due to the close retention time and fragmentation pattern. Marker 3 had fragmentation spectra similar to the other betaines reported. Some chemical structures were proposed based on the amino acid proline, although there are not enough data to propose a definitive structure (Figure 4). Table 3 shows the available literature dealing with the biomarkers related to citrus intake.

Despite having linked all these compounds with orange consumption, it must be emphasized that they are usually exclusive to this type of food. Stachydrine is the most used biomarker as proof of citrus consumption. However, in some populations, it has also been detected as marker for other foods such as tubers of the vegetable *Stachys affinis*, also known as Chinese artichoke [26]. Therefore, previous information of the foods more consumed in each population is important to select the most specific marker of orange intake, or even to use a combination of markers, and not just one, in selected populations. In order to achieve more confidence in tentative identification, a CCS predictor model machine-based in artificial neural network (ANN) developed for protonated ions by our group was employed [45]. The CCS values predicted for protonated ions are shown in Table 2. No prediction for the other adduct or dimers could be made as this tool is not yet developed for them. The relative errors obtained with this prediction tool were below 6% for 95% of all CCS values tested. Nevertheless, the results obtained for the phase II sulfate conjugated metabolites (of synephrine, hesperitine and N-methyltyramine) were between 5% and 7%. This higher error value is probably due to the lack of glucuronides and sulfate metabolites during the building of the CCS prediction model. Compared to other markers, good results were obtained, showing a prediction error below 1% for stachidrine and betonicine and below 3% in de case of N-methyl-proline and Marker 3. The CCS predicted in the two structure proposals for marker 7 did not differ enough to decide between them.Despite having linked all these compounds with orange consumption, it must be emphasized that they are usually exclusive to this type of food. Stachydrine is the most used biomarker as proof of citrus consumption. However, in some populations, it has also been detected as a marker for other foods such as tubers of the vegetable *Stachys affinis*, also known as Chinese artichoke [26]. Therefore, previous information of the foods more consumed in each population is important to select the most specific marker of orange intake, or even to use a combination of markers, and not just one, in selected populations. In order to achieve more confidence in tentative identification, a CCS predictor model machine-based in artificial neural network (ANN) developed for protonated ions by our group was employed [45]. The CCS values predicted for protonated ions are shown in Table 2. No prediction for the other adduct or dimers could be made as this tool is not yet developed for them. The relative errors obtained with this prediction tool were below 6% for 95% of all CCS values tested. Nevertheless, the results obtained for the phase II sulfate conjugated metabolites (of synephrine, hesperitine and N-methyltyramine) were between 5% and 7%. This higher error value is probably due to the lack of glucuronides and sulfate metabolites during the building of the CCS prediction model. Compared to other markers, good results were obtained, showing a prediction error below 1% for stachidrine and betonicine and below 3% in de case of *N*-Methyl-proline and Marker 3. The CCS predicted in the two structure proposals for marker 3 did not differ enough to decide between them. The CCS is used as an additional identification point that allows the identity confirmation. If the CCS is different enough it will be able to distinguish between two isobaric and even isomeric compounds. Nevertheless, there is not enough information and CCS recompilation to apply this differentiation.

The increasing interest in the CCS as an additional molecular descriptor for untargeted metabolomic studies lies in its high capability to improve identification workflows. With IMS, postionization separation is undertaken based on the shape of the compounds and providing CCS as an additional physicochemical. In contrast to other parameters such as retention time, this value is highly reproducible since it is not influenced by the nature of the matrix or the separation employed. Therefore, it is of great importance to report these results, so that they can be included in compound libraries and, in this way, potentially improve the very time-consuming identification step in untargeted metabolomics studies.

Nevertheless, the ideal situation for unequivocally confirming the identity of the reported markers would be a comparison with a reference standard. Unfortunately, many of them are not commercially available, or are very expensive.

## 4. Conclusions

The potential of the untargeted metabolomics approach, along with the novelty of UHPLC-IMS-QTOF instruments for food intake biomarkers has been demonstrated with the tentative elucidation of 7 short-term markers of orange consumption (Clementines) in human plasma: Stachydrine, betonicine, *N*-methyl-proline, as well as phase II sulfate conjugate of synephrine, *N*-methyltyramine and hesperitin (only reported as biomarker of citrus intake in urine). Moreover, the recompilation in databases of CCS as an additional molecular descriptor, independent from the sample matrix nature and separation technique, will facilitate the future compound identification process. The markers have turned out to be indicators of citrus intake, not just orange. However, oranges (mainly *Citrus reticulata* and *Citrus sinensis*) are the fruits most consumed in the citrus group). The combined use of two or more of these markers can contribute to increase the specificity of the citrus intake.

## Figures and Tables

**Table 1:** Demographic, anthropometric and clinical characteristics of the participants by sex.

Characteristics	Total (*n* = 30)	Men (*n* = 22)	Women (*n* = 8)
Age (years)	25.0 ± 0.5	25.4 ± 0.6	23.8 ± 0.9
BMI (Kg/m^2^)	25.0 ± 1.0	25.8 ± 1.3	22.6 ± 1.1
SBP (mm Hg)	124 ± 3	129 ± 3	111 ± 3
DBP (mm Hg)	74 ± 2	75 ± 2	74 ± 2
Total cholesterol (mg/dL)	186.7 ± 5.9	191.4 ± 6.8	172.3 ± 11.6
LDL-C (mg/dL)	121.5 ± 4.6	126.8 ± 5.0	104.9 ± 8.8
HDL-C (mg/dL)	57.5 ± 2.7	53.6 ± 2.7	68.2 ± 5.4
Triglycerides (mg/dL)	75.4 ± 7.5	83.6 ± 9.7	52.8 ± 3.4
Fasting glucose (mg/dL)	86.7 ± 1.1	88.2 ± 1.3	82.7 ± 1.6

Values are mean ± SE for continuous variables. BMI indicates body mass index; SBP indicates Systolic Blood Pressure, DBP indicates Diastolic Blood Pressure; LDL-C indicates Low-Density Lipoprotein cholesterol; HDL-C indicates High-Density Lipoprotein cholesterol.

**Table 2:** Compound list obtained from the untargeted metabolomic approach for determining acute biomarkers of orange consumption.

No.	Compound	Elemental Composition	P[corr]	Feature	Rt (min)	Experimental Neutral Mass (Da)	Theoretical Neutral Mass (Da)	Mass Error (mDa/ppm)	CCS (Å^2^) de/protonated Molecule *m*/*z*	Predicted CCS (Å^2^) Protonated Molecule *m*/*z*^1^	CCS Delta Error (%)	Adducts Detected
1	Synephrime hydrogen sulfate	C_9_H_13_NO_5_S	0.64	HIPOS_2.17_247.0510n	2.17	247.0510	247.0514	−0.4/−1.6	158.27	149.79	−5.36	[M+H]^+^ [M-H2O+H]^+^ [2M+H]^+^ [2M+Na]^+^
			0.86	HINEG_2.22_246.0434m/z	2.22	247.0507	247.0514	−0.7/−2.8	155.40	---	---	[M-H]^−^ [(M-H+Na)-Cl]^−^ [2M-H]^−^ [2M-2H+Na]^−^
2	N-methyltyramine hydrogen sulfate	C_9_H_13_NO_4_S	0.63	HIPOS_1.89_232.0629m/z	1.89	231.0556	231.0565	−0.9/−3.9	154.00	146.16	−5.09	[M+H]^+^
			0.82	HINEG_1.89_230.0483m/z	1.89	231.0556	231.0565	−0.9/−3.9	154.49	---	---	[M-H]^−^
3	Hesperitin hydrogen sulfate	C_16_H_14_O_9_S	0.61	HINEG_0.58_381.0279m/z	0.58	382.0352	382.0359	−0.7/−1.8	175.63	---	---	[M-H]^−^
			0.57	HIPOS_0.57_383.0430m/z	0.57	382.0357	382.0359	−0.2/−0.52	172.50	186.02	−7.83	[M+H]^+^
4	N-methyl-proline	C_6_H_11_NO_2_	0.84	HIPOS_4.73_130.0856m/z	4.73	129.0783	129.0790	−0.7/−5.4	127.17	124.4	−2.18	[M+H]^+^
5	Betonicine	C_7_H_13_NO_3_	0.93	HIPOS_4.79_159.0890n	4.79	159.0890	159.0895	−0.5/−3.1	129.02	130.25	+0.95	[M+H]^+^ [M+Na]^+^ [M+K]^+^ [2M+Na]^+^
			0.92	RPPOS_0.66_160.0970m/z	0.66	159.0897	159.0895	+0.2/+1.3	129.02	130.25	+0.95	[M+H]^+^ [M+Na]^+^
6	Stachydrine	C_7_H_13_NO_2_	0.92	HIPOS_5.02_143.0941n	5.02	143.0941	143.0946	−0.5/−3.5	127.17	127.55	+0.30	[M+H]^+^ [M+Na]^+^ [M+K]^+^ [2M+H]^+^ [2M+Na]^+^
			0.96	RPPOS_0.64_143.0947n	0.64	143.0947	143.0946	+0.1/+0.7	127.18	127.55	+0.29	[M+H]^+^ [M+Na]^+^
7	unknown	C_6_H_11_NO_3_	0.65	HIPOS_2.76_146.0806m/z	2.76	145.0733	145.0739	−0.6/−4.1	123.81	127.11/127.03	+2.66/+2.60	[M+H]^+^

**Table 3:** Literature found on biomarkers related with the citrus intake.

No.	Compound	Related Compounds Found in the Literature	Citrus Fruit and/or Product Consumed	Bio Fluid Sample	Analysis Platform	Literature on Detection as Metabolite	Literature Demonstrating the Presence in Citrus
1	Synephrime hydrogen sulfate	Synephrine and/or other phase I/II metabolites	orange juice	urine	LC-MS/MS LC-ECD	[30,52]	[49,53]
2	N-methyltyramine hydrogen sulfate	N-methyltyramine and/or other phase I/II metabolites	orange, grapefruit, orange juice	urine	LC-HRMS	[54]	[49,53]
3	Hesperitin hydrogen sulfate	Hesperitin and/or other phase I/II metabolites	orange, grapefruit, orange juice	urine	LC-HRMS LC-MS/MS	[17,54]	[55]
4	N-methyl-proline		orange juice	urine	LC-HRMS	[21,25,27,28]	[56]
5	Betonicine		orange, grapefruit, orange juice	plasma, urine	FIA-HRMS LC-HRMS LC-MS/MS	[17,19,25,28,54]	[56]
6	Stachydrine		orange, grapefruit, orange juice, grapefruit juice	plasma, serum, urine	FIA-HRMS LC-M/MS LC-HRMS 1H NMR	[16,17,18,19,20,21,23,24,25,27,28,54]	[56]
7	Unknown	NA	NA	NA	NA	NA	NA

Liquid chromatography coupled to tandem mass spectrometry (LC-MS/MS); LC coupled to electrochemical detection (LC-ECD); LC coupled to high-resolution mass spectrometry (LC-HRMS); Flow injection analysis coupled HRMS (FIA-HRMS) and; proton nuclear magnetic resonance (1H NMR)

## Supplementary Materials

The following are available online at https://www.mdpi.com/2072-6643/12/7/1916/s1, Figure S1: PCA score plot component 1 vs. component 2 of HILIC in negative ionization mode, explaining the 41.8% and 14.7% of the variance; Figure S2: PCA score plot component 1 vs. component 2 of RP in positive ionization mode, explaining the 47.1% and 11.7% of the variance; Figure S3: PCA score plot component 1 vs. component 2 of RP in negative ionization mode, explaining the 28.5% and 26.7% of the variance; Figure S4: OPLS-DA score plot based on the features with repeated measures ANOVA *p*-value ≤ 0.05, where the 94% of the total variance is explained; Figure S5: PCA score plot component 1 vs. component 2 obtained with the 43 features highlighted in the OPLS-DA with a P[corr] ≥ |0.6|, explaining the 79.3% and 9.6% of the variance; Figure S6: Extraction ion chromatograms of ions present in the LE function of HILIC pos and HILIC neg analysis.
