# Supplementary material for: Ultra-Performance Liquid Chromatography-Ion Mobility Separation-Quadruple Time-of-Flight MS (UHPLC-IMS-QTOF MS) Metabolomics for Short-Term Biomarker Discovery of Orange Intake: A Randomized, Controlled Crossover Study
Source: Nutrients. 2020 Jun 29;12(7):1916. doi: 10.3390/nu12071916 (PMC7400617; doi:10.3390/nu12071916)

ONLINE SUPPORTING MATERIAL

Ultra-Performance Liquid Chromatography-Ion Mobility Separation-Quadruple Time-of-Flight MS (UHPLC-IMS-QTOF MS) Metabolomics for Short-Term Biomarker Discovery of Orange Intake: A Randomized, Controlled Crossover Study

Leticia Lacalle-Bergeron, Tania Portolés, Francisco J. López, Juan Vicente Sancho, Carolina Ortega-Azorín, Eva M. Asensio, Oscar Coltell and Dolores Corella

Figure S1 2
Figure S2 3
Figure S3 4
Figure S4 5
Figure S5 6
Figure S6 7

Figure S1. PCA score plot component 1 vs component 2 of HILIC in negative ionization mode, explaining the 41.8% and 14.7% of the variance, respectively. The purple points (14 QC samples) are grouped and centered in the plot. 4 plasma samples were obtained from each of the 30 participants, obtaining 30 samples per group. 120 plasma samples were analyzed in total. The samples obtained at t = 0 h and t = 4 h after the intake of an isocaloric beverage (IB) and the samples obtained at t = 0 h and t = 4 h after orange intake are colored in green, blue, black and orange, respectively.

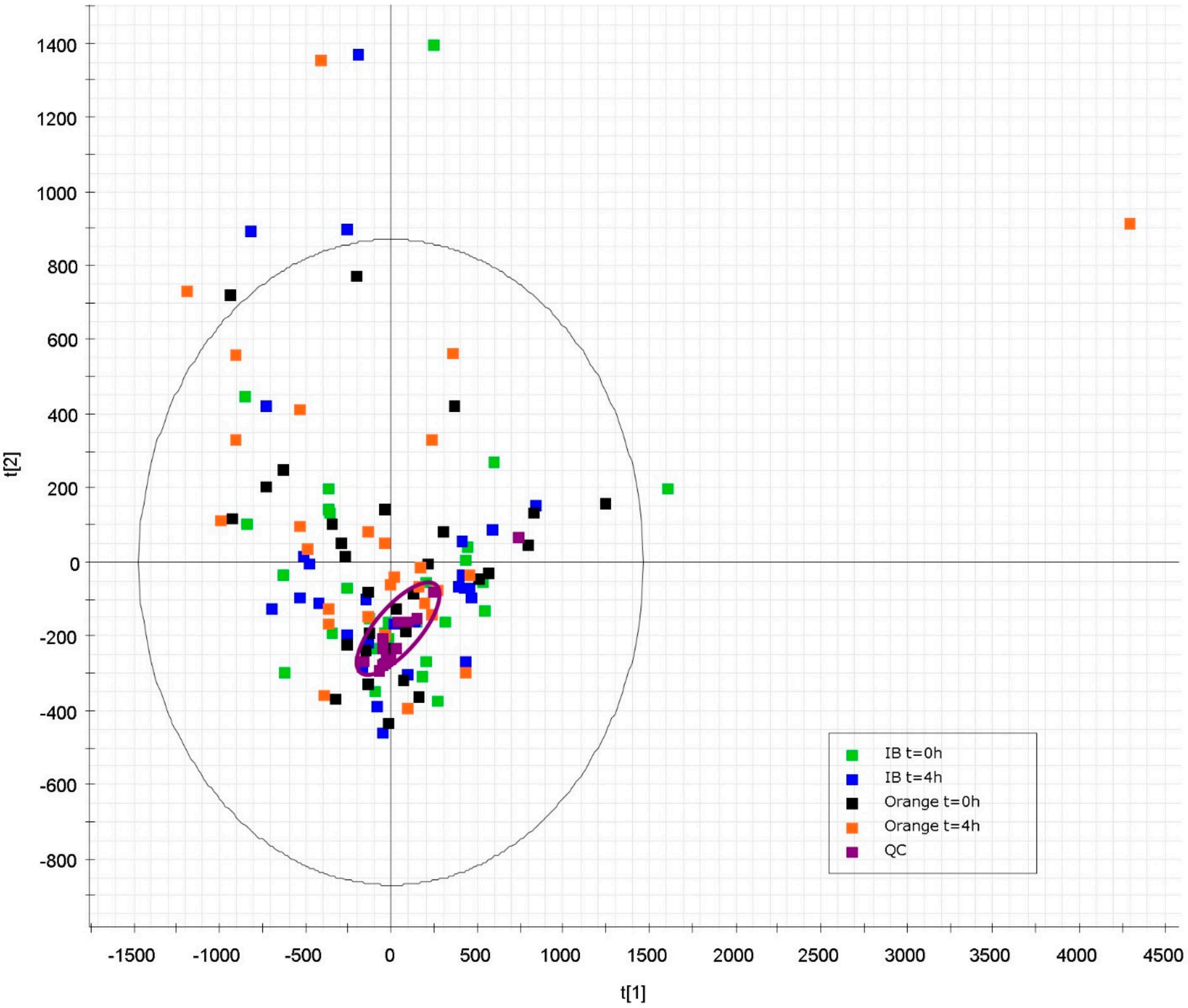

Figure S2. PCA score plot component 1 vs component 2 of RP in positive ionization mode, explaining the 47.1% and 11.7% of the variance, respectively. The purple points (14 QC samples) are grouped and centered in the plot. 4 plasma samples were obtained from each of the 30 participants, obtaining 30 samples per group. 120 plasma samples were analyzed in total. The samples obtained at t = 0 h and t = 4 h after the intake of an isocaloric beverage (IB) and the samples obtained at t = 0 h and t = 4 h after orange intake are colored in green, blue, black and orange, respectively.

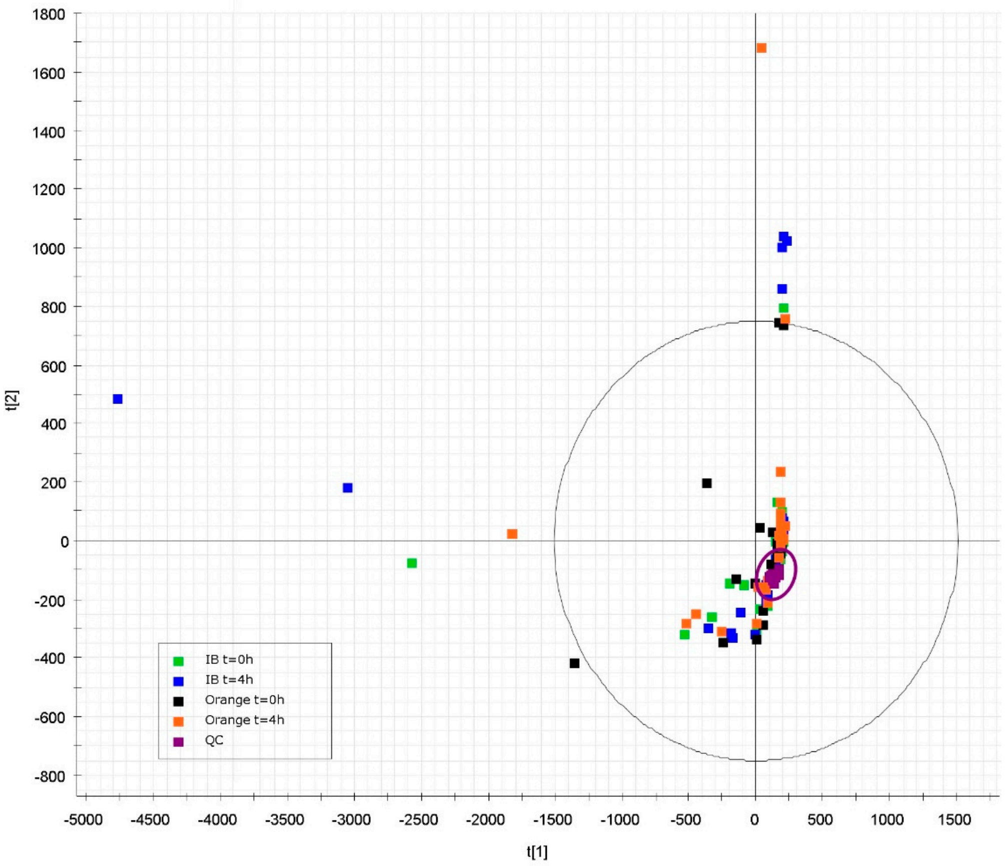

Figure S3. PCA score plot component 1 vs component 2 of RP in negative ionization mode, explaining the 28.5% and 26.7% of the variance, respectively. The purple points (14 QC samples) are grouped and centered in the plot. 4 plasma samples were obtained from each of the 30 participants, obtaining 30 samples per group. 120 plasma samples were analyzed in total. The samples obtained at t = 0 h and t = 4 h after the intake of an isocaloric beverage (IB) and the samples obtained at t = 0 h and t = 4 h after orange intake are colored in green, blue, black and orange, respectively.

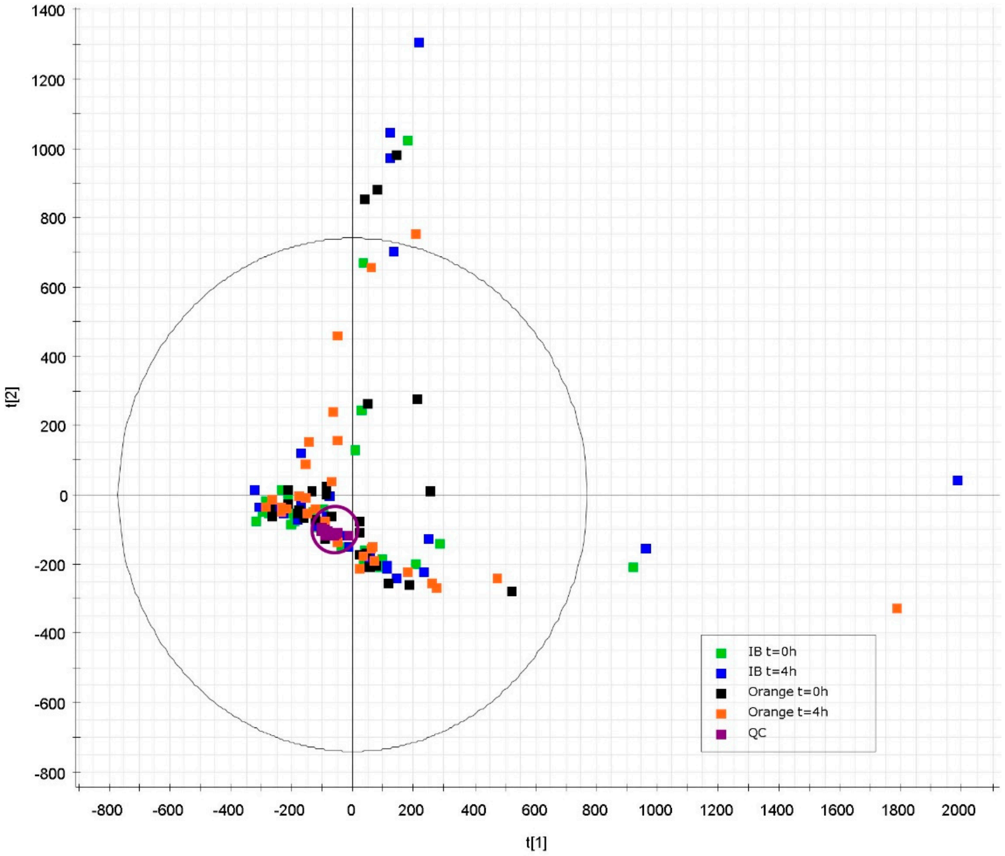

Figure S4. OPLS-DA score plot based on the features with repeated measures ANOVA *p*-value ≤ 0.05, where the 94% of the total variance is explained. The samples obtained at t = 0 h and t = 4 h after the intake of an isocaloric beverage (IB) and the samples obtained at t = 0 h are marked as "others" group and colored in black; while samples obtained t = 4 h after orange intake are colored in orange.

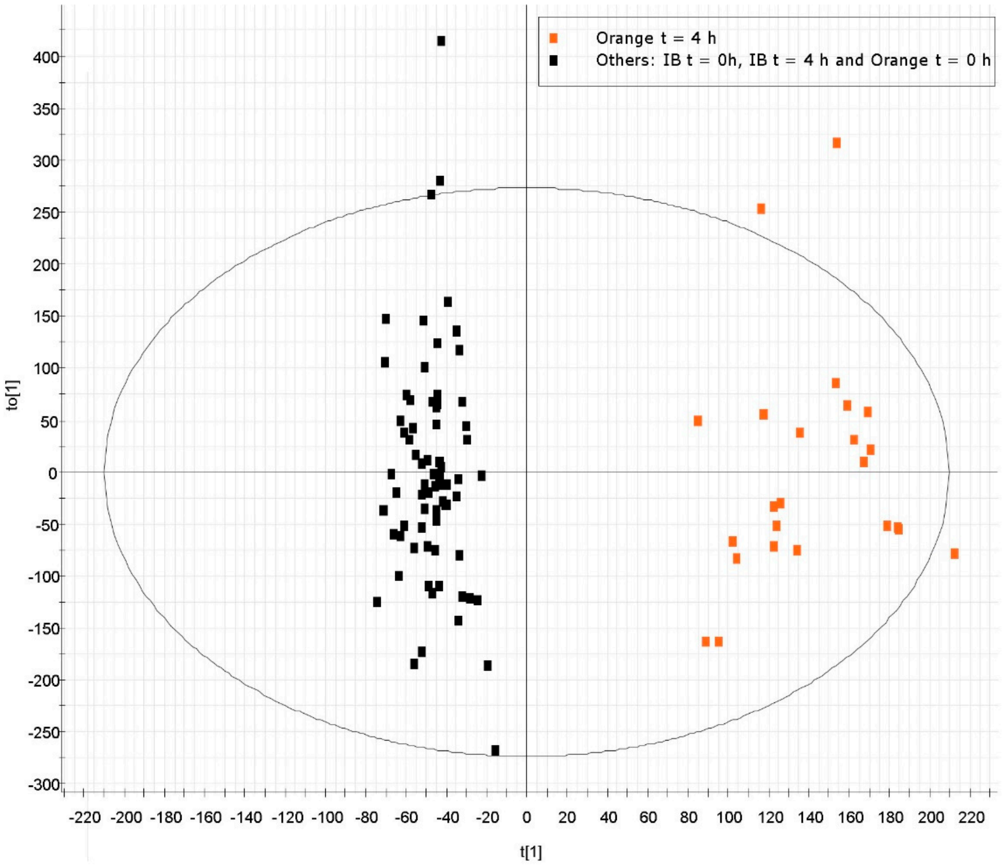

Figure S5. PCA score plot component 1 vs component 2 obtained with the 43 features highlighted in the OPLS-DA with a P[corr] ≥ |0.6|, explaining the 79.3% and 9.6% of the variance, respectively. The samples obtained at t = 0 h and t = 4 h after the intake of an isocaloric beverage (IB) and the samples obtained at t = 0 h and t = 4 h after orange intake are colored in green, blue, black and orange, respectively.

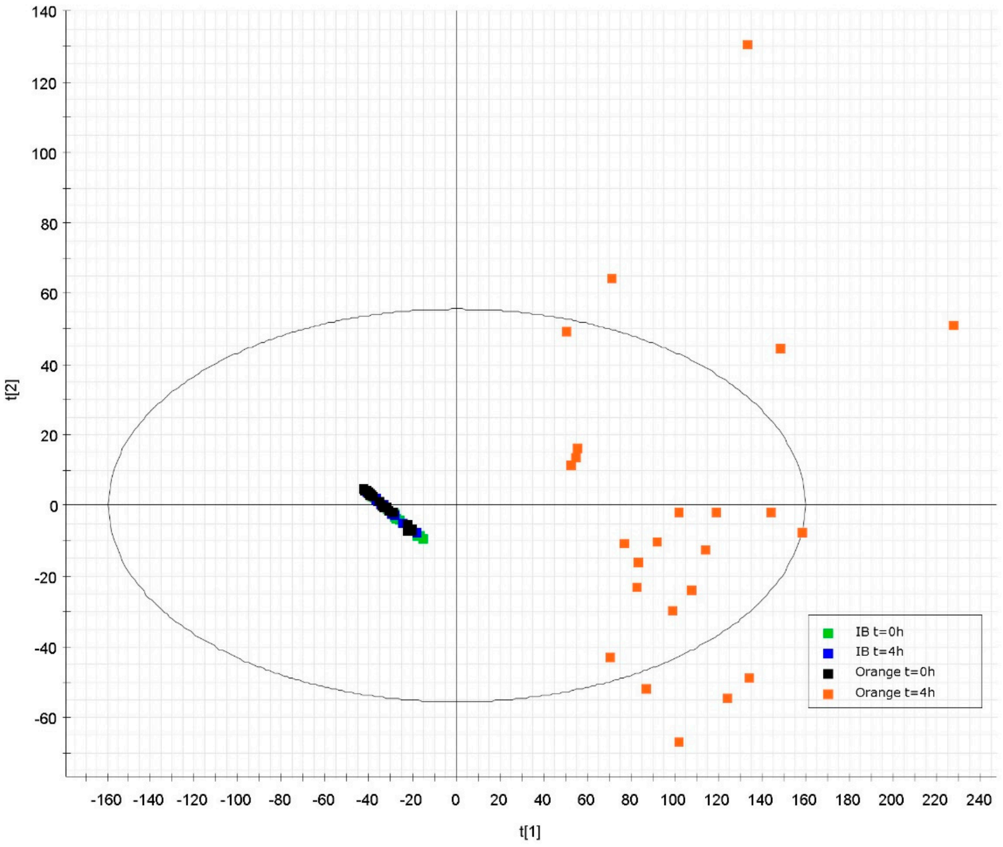

Figure S6. Extraction ion chromatograms of ions present in the LE function of HILIC pos and HILIC neg analysis. The experimental RT and CCS are shown.

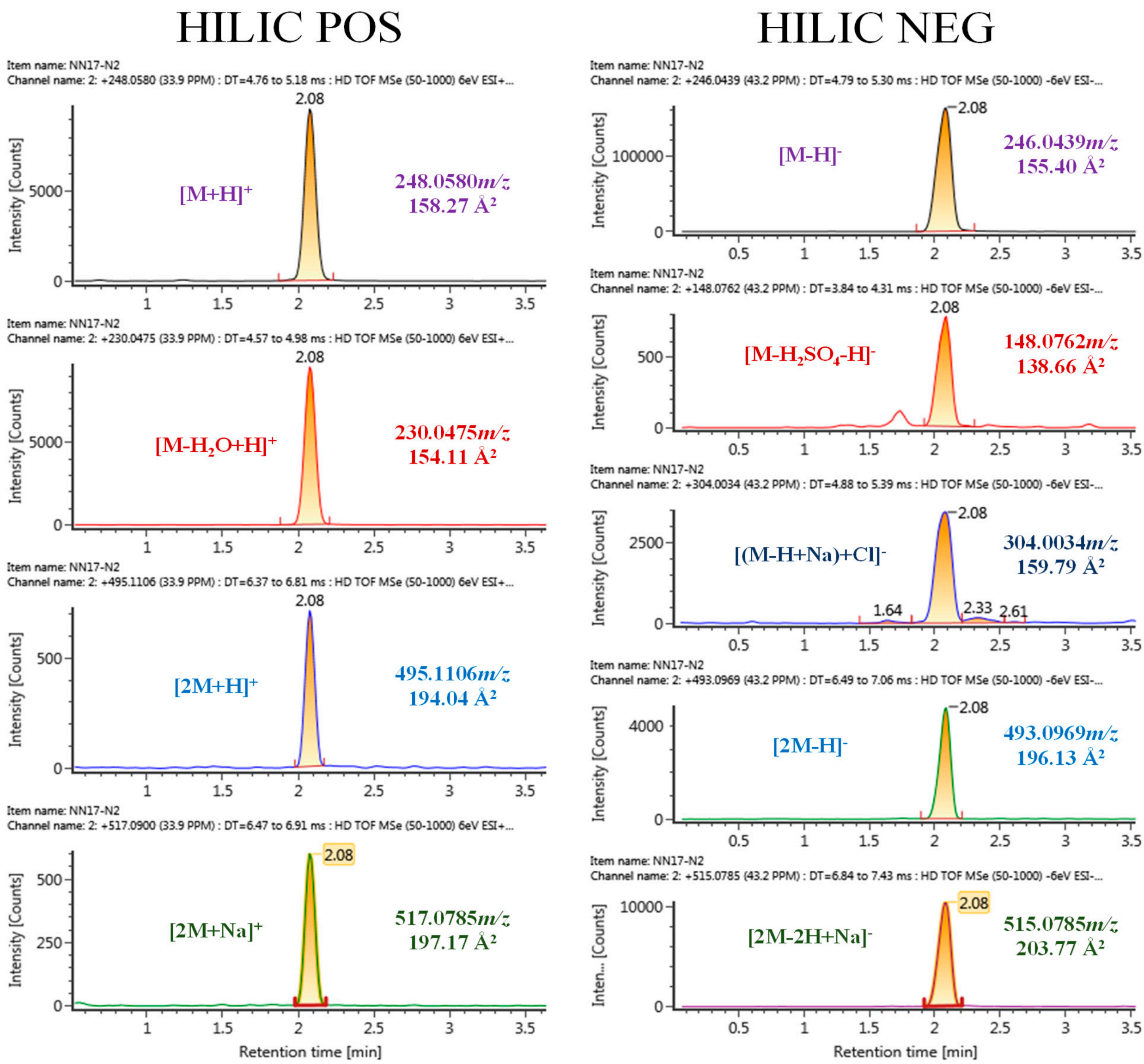

Supplement: Supplementary file 1 [file nutrients-12-01916-s001.pdf]
